# Notch Ligand Delta-Like 4-Pretreated Dendritic Cells Alleviate Allergic Airway Responses by Enhancing IL-10 Production

**DOI:** 10.1371/journal.pone.0063613

**Published:** 2013-05-16

**Authors:** Huei-Mei Huang, George Hsiao, Chia-Kwung Fan, Chu-Lun Lin, Sy-Jye Leu, Bor-Luen Chiang, Yueh-Lun Lee

**Affiliations:** 1 Graduate Institute of Medical Sciences, College of Medicine, Taipei Medical University, Taipei, Taiwan; 2 Department of Pharmacology, College of Medicine, Taipei Medical University, Taipei, Taiwan; 3 Department of Parasitology, College of Medicine, Taipei Medical University, Taipei, Taiwan; 4 Department of Microbiology and Immunology, College of Medicine, Taipei Medical University, Taipei, Taiwan; 5 Graduate Institute of Clinical Medicine, College of Medicine, National Taiwan University, Taipei, Taiwan; French National Centre for Scientific Research, France

## Abstract

The Notch pathway plays a role in the processes of cell proliferation, differentiation, and apoptosis, which affect the development and function of various organs. Dendritic cells (DCs), as professional antigen-presenting cells (APCs), induce T cell activation and promote T cell differentiation by antigen stimulation. Research has shown that Notch ligand delta-like 4 (Dll4) in APCs is associated with stimulation of a Th1-type response. However, the regulatory roles of Dll4 in the activation and function of DCs have yet to be clearly elucidated. In this study, we demonstrated that activation of Dll4-pretreated bone marrow-derived DCs by performing ovalbumin (OVA) stimulation expressed a high level of interleukin (IL)-10 without diminishing IL-12 production. By contrast, the proinflammatory cytokines, IL-1β, IL-6, and tumor necrosis factor (TNF)-α, decreased in Dll4-pretreated DCs by performing either lipopolysaccharide (LPS) or OVA stimulation. Compared to fully mature DCs, lower levels of MHC class II CD40 and higher levels of CD80 and CD86 molecules were expressed in these semi-mature like DCs. Dll4 Notch signaling also enhanced Notch ligand mRNA expression of Dll1, Dll4, and Jagged1 in DCs. Dll4-modified DCs exhibited a reduced capacity to stimulate the proliferation of OVA-specific CD4^+^ T cells, but actively promoted large amounts of IL-10 production in these activated T cells. Furthermore, immunomodulatory effects of Dll4-modified DCs were examined in an established asthmatic animal model. After adoptive transfer of OVA-pulsed plus Dll4-pretreated DCs in OVA-immunized mice, OVA challenge induced lower OVA-specific immunoglobulin E (IgE) and higher IgG_2a_ antibody production, lower eotaxin, keratinocyte-derived chemokine (KC), IL-5, and IL-13 release in bronchial alveolar lavage fluid, attenuated airway hyper-responsiveness, and promoted higher IL-10 and interferon (IFN)-γ production in the spleen. In summary, our findings elucidate the new role of Dll4 in the phenotype and function of DCs and provide a novel approach for manipulating T cell-driven deleterious immune diseases.

## Introduction

Dendritic cells (DCs) are the most potent antigen (Ag)-presenting cells (APCs) of the immune system and are critically involved in initiating primary immune responses and inducing T cell responses. Immature DCs display the highest capacity to internalize Ags but are poor T-cell activators. After Ag uptake, DCs mature, become specialized in Ag presentation, and are excellent T-cell stimulators. Maturation of DCs can be triggered by components of the bacterial wall, proinflammatory cytokines, and viral components [1]. As mature APCs, DCs display long-lasting peptide-MHC class II complexes on the surface and upregulate surface levels of co-stimulatory molecules (CD40, CD80, and CD86) and intercellular adhesion molecules (CD54). Additionally, mature DCs produce high levels of interleukin (IL)-12 and tumor necrosis factor (TNF)-α, and ultimately activate T cells [2].

Vertebrates possess 4 identified Notch receptors (Notch 1 to 4) and 5 ligands of the Delta-like (Delta-like1 [Dll1], Dll3, and Dll4) and Jagged (Jgd) families (Jgd1 and Jgd2). Notch receptors and ligands are expressed in developing and mature lymphocytes and lymphoid tissues, and have been shown to play crucial roles in T-B lineage commitment and thymic T cell development [3]–[7]. In addition to this involvement in lymphopoiesis, it has been well established that Notch ligands influence T cell differentiation, in that the Dll group directs T cell polarization toward T helper type 1 (Th1), whereas the Jgd group promotes Th2 or regulatory T (Treg) responses [8]–[13]. Notch receptors and their ligands are both expressed in DCs, and the role of Notch signaling in DC differentiation has been explored. It appears that Dll1 promotes generation of fully differentiated DCs, whereas Jgd1 prevents their transition to terminally differentiated DCs [14]–[16]. Unstimulated DCs express low levels of Dll and Jgd. Under different conditions, expression of Notch ligands can be induced toward preferential expression of either Dll or Jgd. Previous studies have recently established that pathogen-derived Th1-promoting stimuli (i.e., lipopolysaccharide; LPS) induce Dll4 expression, whereas Th2-promoting stimuli (i.e., cholera toxin) induce Jgd2 expression by DCs [17]–[20]. By contrast, much less is known about the effects of Notch on the maturation and function of DCs. One study reported that a co-culture of monocyte-derived human DCs with cells expressing Jgd1 induced IL-12 production, T cell proliferative responses, and interferon (IFN)-γ production [21]. However, recent studies have indicated that DCs matured by immobilized Jgd1 expressed a high level of IL-10 and promoted the expansion of Treg cells [22].

Allergic asthma is a disease characterized by elevated serum immunoglobulin E (IgE), airway hyper-responsiveness (AHR), and airway inflammation. Previous studies have shown that CD4^+^ Th2 cells and their cytokines (IL-4, IL-5, and IL-13) are responsible for initiating and maintaining allergic disorders. Consistent with the view that Dll promotes Th1 responses, Dll4-Fc fusion proteins have been shown to promote Th1 cell development [23]. Additionally, by using Dll4 expressing DCs, Sun et al. revealed the ability of Dll4 to promote Th1 cell development and used them to strongly inhibit Th2 development [24]. Thus, based on this evidence, we hypothesized that induction of high levels of expression of Dll4 in Dll4-pretreatd DCs may be a potential strategy for inhibiting Th2 immune responses.

In the current study, we characterized the influence of Notch ligand Dll4 pretreatment following antigen stimulation on the phenotype and function of DCs. We demonstrated the ability of Dll4 to induce a high level of IL-10 expression and inhibit the production of proinflammatory cytokines in ovalbumin (OVA)-stimulated DCs. In addition, these Dll4-modulated DCs displayed a semi-mature like phenotype, and their capacity to induce T cell proliferation was impaired. Furthermore, OVA-specific T cells cultured using Dll4-pretreated DCs produced high levels of IL-10 and IFN-γ. More important, adoptive transfer of these DCs suppressed allergic airway inflammation in a murine model of OVA-induced asthma. These data imply that Dll4 can alter DC activation and function to promote IL-10 production by activated T cells, and converts T cells with regulatory activity in Th2-driven immune diseases.

## Materials and Methods

### Mice

Female BALB/c and DO11.10 mice expressing a transgenic T-cell receptor that is specific for amino acids 323 to 339 of OVA were purchased from the Animal Center of National Taiwan University (Taipei, Taiwan) and maintained in the Animal Center of Taipei Medical University. All of the mice in this study were between 5 and 10 weeks of age and were age-matched for each experiment. Animal care and handling protocols were approved by the Animal Committee of the College of Medicine, Taipei Medical University.

### Preparation of Bone Marrow-derived DCs

Tibial and femoral bone marrow from 5- to 6-week-old BALB/c mice was isolated. Bone marrow cells were cultured in RPMI-1640 and a 5% fetal bovine serum (FBS) complete medium with IL-4 (1000 U/mL) and GM-CSF (500 U/mL) on Day 0. Every second day, a fresh medium containing GM-CSF and IL-4 was used to replace the old medium, and non-adherent cells were transferred to a new plate to decrease contamination by macrophages.

### Cell Viability Assay

Cell viability was determined using the mitochondrial-dependent reduction of MTT to formazan. Mouse recombinant Dll4 protein (R&D Systems, Minneapolis, MN, USA), which was derived from a murine myeloma cell line, was used as a Notch receptor ligand. To obtain immobilized Dll4, plates were coated with Dll4 in phosphate buffered saline (PBS) overnight at 4°C. On Day 4 of culture, bone marrow-derived DCs were harvested and treated with concentrations of 1 and 5 µg/mL of immobilized Dll4 until the end of culture. On Day 6, DCs were stimulated with or without 100 µg/mL OVA (grade V; Sigma-Aldrich, St-Louis, MI, USA) for an additional 24 h. Untreated DCs were pulsed with OVA or medium alone as the controls. After incubation, cells were collected and seeded onto 96-well plates at 1×10^5^ cells/well. The cells were incubated with 10 µl of 5 mg/mL of MTT for another 4 h, and then solubilized in DMSO. The amount of reduction was measured using a microplate reader at 570 nm.

### Reverse Transcription (RT) and Quantitative Polymerase Chain Reaction (qPCR)

On Day 4 of culture, bone marrow-derived non-adherent cells were collected, washed in Hank’s balanced salt solution (HBSS), and incubated in the presence of immobilized recombinant Dll4 (at 1 and 5 µg/mL) until the end of culture. On Day 6, DCs were stimulated with 100 µg/mL OVA for an additional 5 h. Additionally, 25 µg/mL blocking anti-Dll4 monoclonal antibody (BioLegend, San Diego, CA, USA) was added under culture conditions of pulsed-OVA plus Dll4 (5 µg/mL)-pretreated DCs. Untreated DCs were stimulated with OVA or medium alone as controls. After incubation, cells were collected and total RNA (2 to 4×10^6^ cells) was extracted according to the TRIzol method (Sigma-Aldrich). Complementary DNA (cDNA) was generated from 2 µg of total RNA using a high-capacity cDNA RT kit (Applied Biosystems, Foster City, CA, USA). Four microliters of cDNA were used for PCR master mix and TaqMan assays (Applied Biosystems). qPCR detection of mouse GAPDH, IL-10, IL-12, Dll1, Dll4, Jgd1, and Jgd2 was conducted in triplicate using an Applied Biosystems 7900 PCR system. Cycle thresholds obtained were normalized to GAPDH. Relative multiples of change were calculated using the comparative threshold cycle (C_T_) method, 2^−△△CT^.

### Determination of Cytokine Levels

On Day 6, Dll4-pretreated DCs were stimulated with 100 µg/mL OVA or 100 ng/mL LPS (Sigma-Aldrich) for 24 h to achieve activation before use in the cytokine or phenotypical assays. In the experiments, untreated DCs were pulsed with LPS, OVA, or medium alone as the control. Levels of IL-1β, IL-4, IL-5, IL-6, IL-10, IL-12, TNF-α, and IFN-γ in the culture supernatants from DCs or T cells were assayed by enzyme-linked immunosorbent assay (ELISA) kits (Duoset, R&D Systems, Minneapolis, MN, USA). Quantities of eotaxin, keratinocyte-derived chemokine (KC), IL-5, IL-10, IL-13, and IFN-γ in bronchoalveolar lavage fluid (BALF) and culture supernatants of splenocytes were also evaluated using commercially available ELISA kits (Duoset, R&D Systems).

### Flow Cytometric Analysis of DCs

DCs were stained with fluorescein isothiocyanate (FITC)-conjugated anti-CD11c, anti-CD40, anti-CD54 and anti-CD86, or phycoerythrin (PE)-conjugated, anti-I-A/I-E (MHC class II), anti-CD80, and anti- CD11c (eBioscience, Inc., San Diego, CA, USA) at 4°C for 30 min. The cells were washed and suspended in 0.5 mL of PBS with 0.1% sodium azide. Staining with isotype control antibodies was performed in all experiments. A FACSort cell analyzer (Becton Dickinson, Mountain View, CA, USA) was used for analytical flow cytometry, and data were processed using Cellquest software (Becton Dickinson).

### Isolation of CD4^+^ T cells

Spleens were harvested from female DO11.10 mice and ground into single-cell suspensions. After depleting red blood cells via an ACK lysis buffer, splenocytes were washed 3 times with HBSS and resuspended with MACS buffer (0.5% BSA and 2 mM EDTA in 1x PBS solution) at 10^7^/90 µL cells. Subsequent staining with anti-CD4 magnetic beads (Miltenyi Biotec, Auburn, CA) at 10^7^/10 µL cells was performed for 10 min at 4°C. According to manufacturer instructions, cells were then washed and resuspended in MACS buffer to purify naive CD4^+^ T cells. Positively selected cells were collected for further analysis.

### In vitro Stimulatory Ability of DCs

On Day 7, irradiated OVA-stimulated DCs, OVA/Dll4 co-cultured DCs, and non-treated DCs were harvested for T cell proliferation assay. Freshly isolated DO11.10 CD4^+^ T cells (2×10^5^ cells/well) were co-cultured at these irradiated DCs at DC/T-cell ratios of 1∶5, 1∶10, 1∶20, 1∶40, and 1∶80. Cultures were pulsed with 1 µCi [^3^H] thymidine for the final 18 h of a 72-h culture, and [^3^H] thymidine deoxyribose incorporation was measured using a scintillation counter.

### In vitro Generation of T-cell Lines

To generate T-cell lines, 10^6^ cells/well of naive purified DO11.10 CD4^+^ T cells were co-cultured with immature DCs (control), OVA-activated DCs (DC/OVA), or OVA plus Dll4-treated DCs (DC/OVA-Dll4) at a DC/T-cell ratio of 2: 1 in the presence of IL-2 (10 units/mL). T cells were re-stimulated under the same conditions used for the initial stimulation at 7-d intervals for 2 cycles. For cytokine induction, CD4^+^ T-cell lines (2×10^5^ cells/well) from the third culture were stimulated with 2.5×10^5^ irradiated DCs and doses of OVA_323–339_ peptide of 1, 2, and 4 µg/mL in 96-well plates. Levels of cytokines in the culture supernatants from T-cell lines were assayed after 72 h using ELISA kits.

### Adoptive Transfer Experiments

On Day 6 of culture, Dll4-pretreated (1 and 5 µg/mL) or untreated DCs were cultured with 100 µg/mL OVA protein for an additional 24 h. On Day 7, DCs were harvested, washed, and resuspended in PBS for injection into asthmatic animals. Groups of at least 5 mice were injected intravenously on Days 26 and 37 with 10^6^ Dll4 plus OVA-stimulated DCs (DC/OVA-Dll4) and OVA-stimulated DCs (DC/OVA), respectively. Control mice were administered PBS instead of DCs. On Days 1, 14, and 28, all groups of mice were intraperitoneally immunized with 50 µg of OVA emulsified in 4 mg aluminum hydroxide (Pierce Chemical, Waltham, MA, USA). Then, mice were intranasally challenged with 100 µg OVA on Days 39 and 40. Subsequently, mice were exposed to OVA aerosols (5% OVA in normal saline solution) for 3 consecutive days (Days 41, 42, and 43) for 30 min daily, and AHR was measured 1 d after the final challenge (on Day 44).

### Serum Antibody Assay

Serum samples were collected from the retro-orbital venous plexus after intraperitoneal immunization with the OVA antigen. OVA-specific immunoglobulin E (IgE), IgG_1_ and IgG_2a_ serum antibody titers were determined using ELISA (Becton Dickinson Biosciences, San Jose, CA, USA). Antibody levels were compared with IgE, IgG_1_ and IgG_2a_ standards of predetermined concentrations (IgE at 1 µg/mL, IgG_1_ = 25 µg/mL and IgG_2a_ at 10 µg/mL). The concentration of standard serum was arbitrarily assigned as 1 ELISA unit (1 EU).

### Determination of Airway Responsiveness

At 24 h after the last aerosol exposure, airway function was measured by detecting changes in lung resistance in response to increasing doses of aerosolized methacholine (MCh, Sigma-Aldrich) in anesthetized mice. The mice were anesthetized, tracheostomized, and mechanically ventilated at a rate of 150 breaths/min, with a tidal volume of 0.3 mL and a positive end expiratory pressure of 3 to 4 cm H_2_O using a computer-controlled small-animal ventilator (model 683, Harvard Rodent Ventilator, Mount Holly, NJ, USA). PE-50 tubing was inserted into the esophagus to the level of the thorax and coupled with a pressure transducer. Flow was measured using electronic differentiation of the volume signal, and changes in pressure, flow, and volume were recorded. Pulmonary resistance was calculated using software (model PNM-PCT100 W, LDS PONEMAH Physiology Platform, LDS Gould, Champaign, IL, USA). MCh aerosol was generated with an inline nebulizer and administrated directly through the ventilator. The resistance of the orotracheal tube was subtracted from all airway resistance measurements. Data are expressed as the pulmonary resistance (R_L_). After measuring the pulmonary function parameters, mice were sacrificed, and their tracheas were immediately lavaged via a tracheal cannula with 1 mL of HBSS, which was free of ionized calcium and magnesium. The lavage fluid was cooled on ice and centrifuged (400×g) at 4°C for 10 min. After centrifugation, the BALF was collected for the chemokine and cytokine assays.

### Analysis of the Cellular Composition of Bronchoalveolar Lavage Fluid

One day after measuring the pulmonary function parameters, the mice were sacrificed, and their tracheas were immediately lavaged 3 times via a tracheal cannula with 1 mL of HBSS that was free of ionized calcium and magnesium. The lavage fluid was cooled on ice and centrifuged (400×*g*) at 4°C for 10 min. After washing, the supernatants were collected for the chemokine and cytokine assays, and cell pellets were re-suspended in 1 mL HBSS. The total number of cells in the BALF was counted with a standard hemocytometer. Differential cell counts were performed by counting at least 200 cells in the cytocentrifuged preparations, staining with Liu’s stain solution (Chi I Pao, Taipei, Taiwan), and differentiating according to standard morphological criteria.

### Statistical Analysis

Results are expressed as the mean ± standard error of the mean (SEM). Statistical analysis was performed using one-way analysis of variance (ANOVA) followed by Dunnett’s post hoc test. A p value of <0.05 was considered statistically significant.

## Results

### Dll4 Pretreatment Enhances IL-10 Production and Reduces Proinflammatory Cytokine Secretion in OVA-stimulated DCs

**Figure 1:**
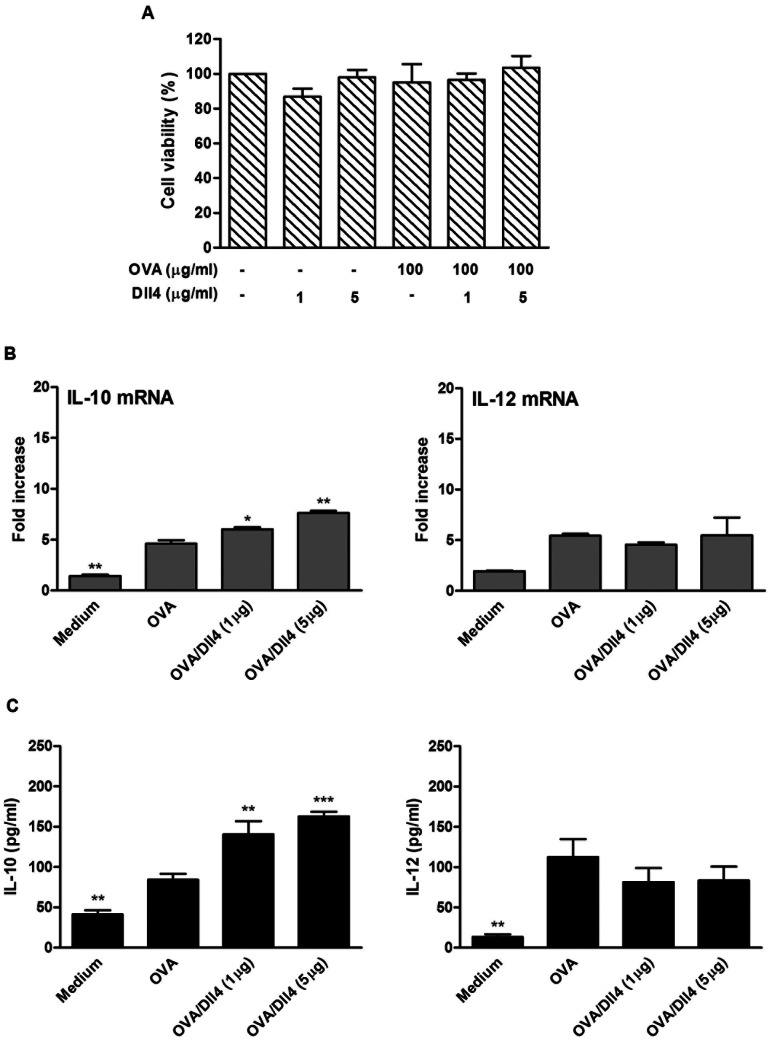
Interleukin-10 expression is upregulated following ovalbumin stimulation in Dll4-pretreated dendritic cells. (A) Cytotoxicity effect of Dll4 on dendritic cells. On Day 4 of culture, bone marrow-derived dendritic cells (DCs) were treated with 1 and 5 µg/mL of Dll4 for 48 h, then cultured with or without 100 µg/mL ovalbumin (OVA) for an additional 24 h. Cell viability was detected using an MTT assay. Untreated DCs were pulsed with OVA or medium alone as the controls. (B) Interleukin (IL)-10 and IL-12 mRNA expression and (C) protein levels from various doses of Dll4-pretreated DCs with OVA stimulation. On Day 4 of culture, bone marrow-derived DCs were treated with medium or various concentrations of Dll4 (1 and 5 µg/mL) for 48 h and then activated by OVA (100 µg/mL) stimulation for an additional 5 or 24 h. Untreated immature DCs served as the control group (DC). Transcripts and protein production of IL-10 and IL-12 were measured using a quantitative real-time RT-PCR and ELISA, respectively. Results from 3 independent experiments are shown and expressed as the mean ± SEM. ** *p*<0.01, *** *p*<0.001 compared to OVA-treated DCs (OVA group).

**Figure 2:**
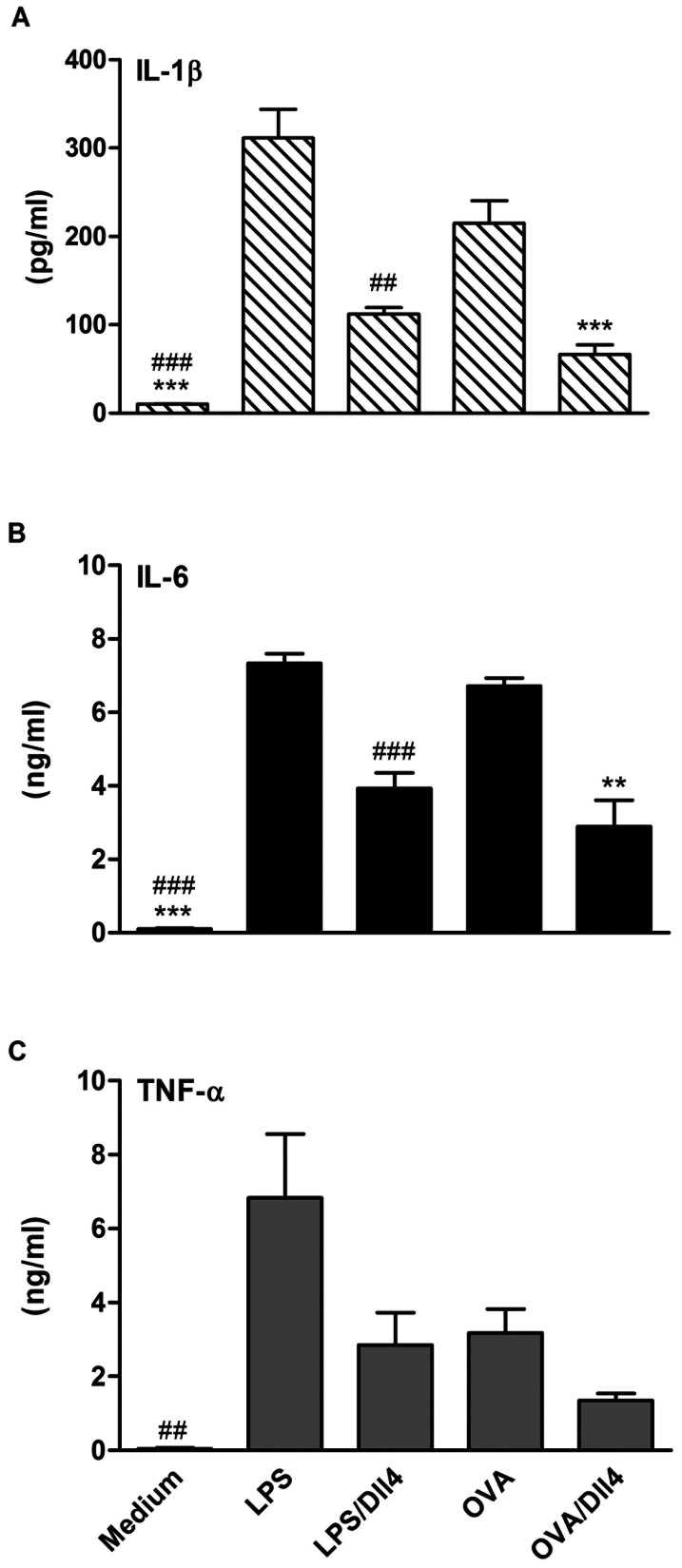
Dll4 pretreatment reduces the production of proinflammatory cytokines in ovalbumin-stimulated dendritic cells. On Day 4 of culture, bone marrow-derived non-adherent cells were treated with medium or Dll4 (5 µg/mL) for 48 h and then activated by lipopolysaccharide (LPS, 100 ng/mL) or ovalbumin (OVA, 100 µg/mL). After 24 h, supernatants were collected and analyzed for interleukin (IL)-1β, IL-6 and tumor necrosis factor (TNF)-α contents by ELISA. Results from 3 independent experiments are shown and expressed as the mean ± SEM. ** *p*<0.01, *** *p*<0.001 compared to OVA-treated DCs (OVA group). ^##^ *p*<0.01, ^###^ *p*<0.001 compared to LPS-treated DCs (LPS group).

Before testing the effects of Dll4 on mouse bone-marrow-derived DCs (BMDCs), we examined the effects of Dll4 protein on cell viability. After 72 h of treatment with 2 doses of immobilized Dll4 (1 and 5 µg/mL) alone or Dll4 followed by OVA stimulation in BMDCs, the cell numbers were counted. The results showed that neither of the concentrations (1 and 5 µg/mL) of Dll4 used in the experiments was cytotoxic (**Figure 1A**). We then addressed the impact of Dll4 on the production of IL-10 and IL-12 cytokines by DCs. BMDCs were exposed to immobilized Dll4 (1 and 5 µg/mL) following OVA stimulation. In the presence of Dll4, increased transcript levels of IL-10 genes were observed in OVA-stimulated DCs (**Figure 1B**). Protein levels of IL-10 also exhibited similarly significant increases in a dose-dependent manner. However, no increase in either IL-12 transcripts or protein levels were detected in DCs pretreated with Dll4 (**Figure 1C**). We then examined the impact of Dll4 on the production of proinflammatory cytokines by ELISA. As depicted in Figure 2, Dll4 obviously inhibited IL-β and IL-6 release by DCs stimulated with either LPS or OVA (**Figure 2**). Similar data was also obtained when TNF-α was quantified.

### Effects of Dll4 on the Maturation and Activation of OVA-stimulated DCs

**Figure 3:**
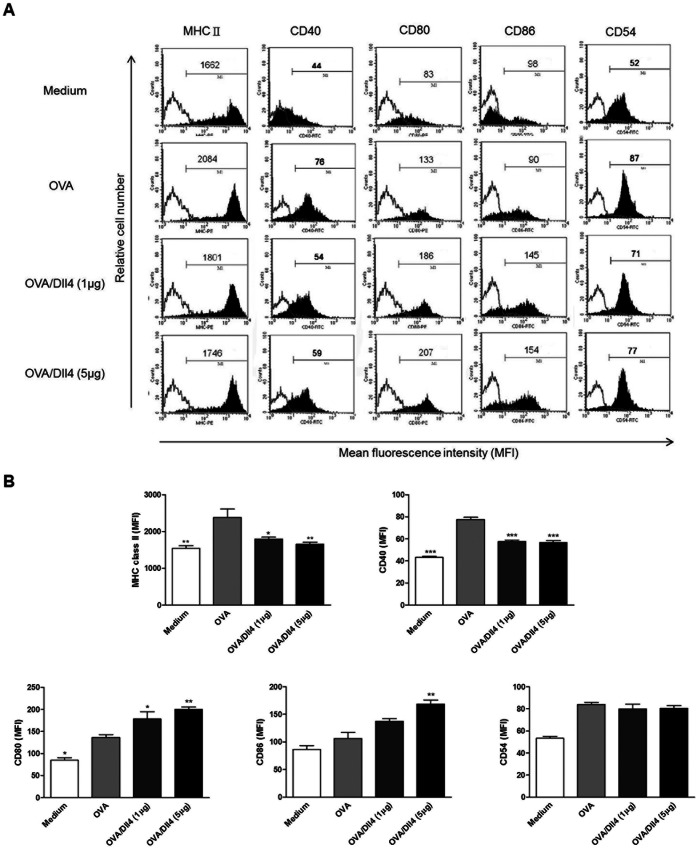
Dll4 pretreatment affects the phenotype of ovalbumin-stimulated dendritic cells. Dll4-pretreated or untreated immature dendritic cells (DCs) were or were not stimulated with ovalbumin (OVA) for 24 h. After incubation, cells were collected, and expression of MHC class II, CD40, CD54, CD80, and CD86 by DCs were analyzed using flow cytometry. DCs stimulated with only OVA (OVA group) were used as the positive control. (A) Values shown in the flow cytometric profiles are the mean fluorescence intensity (MFI). Values shown were from one representative experiment of 3 independent experiments performed. (B) The MFI was calculated, and results are expressed as the mean ± SEM from 3 independent experiments. * *p*<0.05, **p<0.01, ***p<0.001 compared to OVA-treated DCs (OVA group).

To investigate whether Dll4 modulated the maturation and activation of mouse BMDCs in vitro, we compared the phenotype of mouse DCs pretreated with concentrations of immobilized Dll4 of 1 and 5 µg/mL and those left untreated after OVA stimulation. As shown in Figure 3, the upregulation of MHC class II and CD40 molecules during maturation was distinctly reduced in Dll4-pretreated DCs. However, expression of CD80 and CD86 molecules in DCs cultured with Dll4 were significantly higher than those of DCs treated with only OVA (**Figure 3**). Although the difference was not significant, the data indicate that DCs pretreated with Dll4 expressed decreased levels of CD54 molecules compared to untreated DCs. In conclusion, these data indicate that Dll4 pretreatment converted OVA-stimulated DCs to semi-mature like DCs instead of fully mature DCs.

### Dll4 Enhances Jgd1 and Dll mRNA Expression in OVA-stimulated DCs

**Figure 4:**
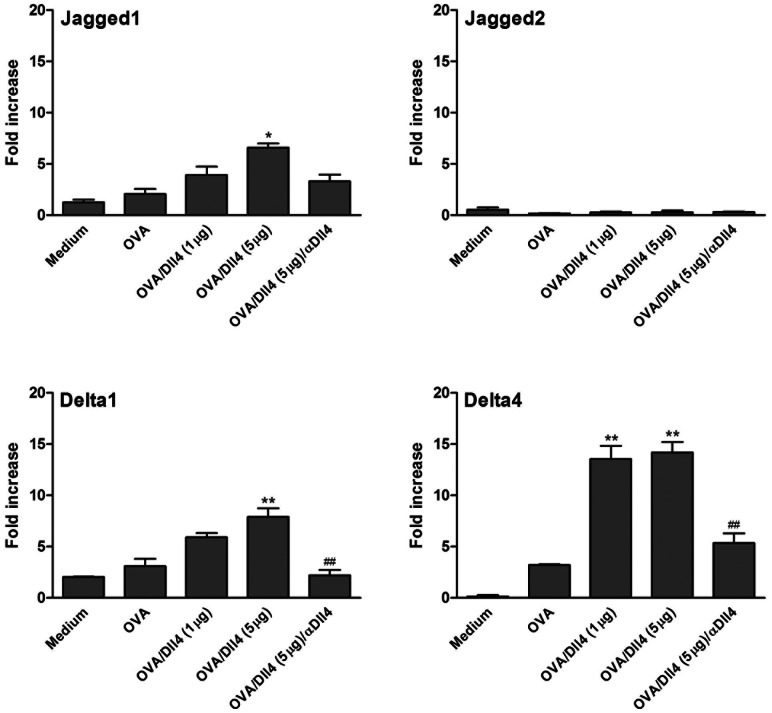
Effects of Dll4 on the expression of Jagged or Delta mRNA in ovalbumin-stimulated dendritic cells. Dll4-pretreated or untreated immature DCs were or were not stimulated with ovalbumin (OVA). Additionally, under culture conditions of Dll4 (5 µg/mL) plus OVA-treated DCs, a blocking anti-Dll4 monoclonal antibody (25 µg/mL) was added until the end of culture. Cells were harvested after 5 h, and Jagged (Jgd)1, Jgd2, Dll1, and Dll4 mRNA accumulations were measured using a real-time RT-PCR. Data are the mean ± SEM and are representative of 3 independent experiments. * *p*<0.05, ** *p*<0.01 compared to OVA-treated DCs (OVA group). ^##^ *p*<0.01 compared to OVA/Dll4 (5 µg) group.

Variations in expression of the Notch ligands, Jgd and Dll, by mature DCs have been shown to be crucial for T cell differentiation. We thus investigated whether Dll4 pretreatment cooperated with or interfered with their expression, as assessed using a real-time RT-PCR. Compared to untreated cells, exposure of DCs to Dll4 following OVA stimulation induced a moderate increase in Jgd1 mRNA synthesis, but no increase in Jgd2 transcripts (**Figure 4**). However, Dll4 stimulation induced higher amounts of Dll1 and Dll4 transcripts in DCs. To ensure that the response is Dll4-specific, a blocking anti-Dll4 antibody was used in this experiment. We observed that the blocking of Dll4-mediated Notch signaling markedly inhibited the mRNA expression of Jd1, Dll1, and Dll4.

### Dll4-pretreated DCs Reduce OVA-specific T cell Activation and Enhance IL-10 and IFN-γ Production by Polarized T cells

**Figure 5:**
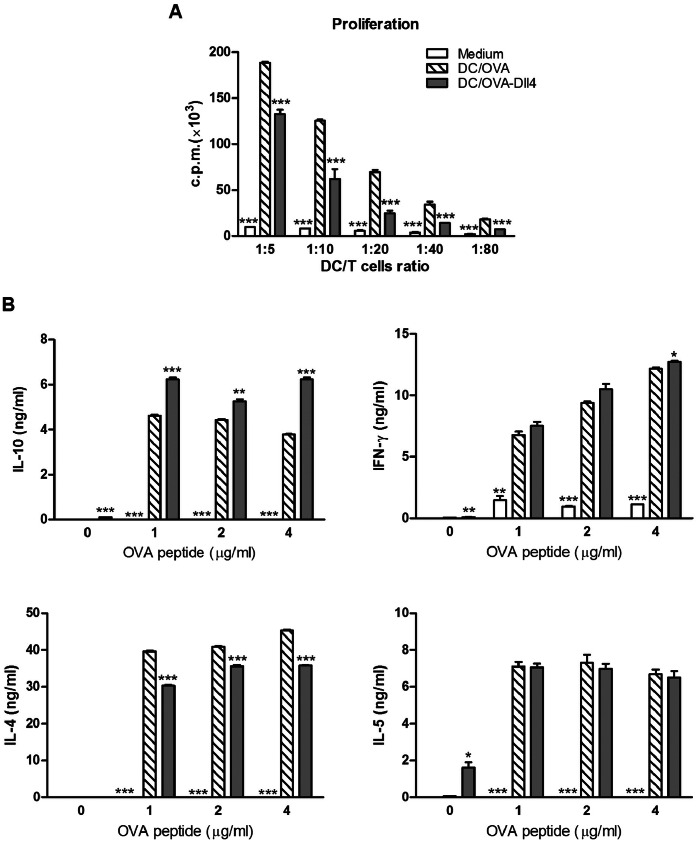
Dll4-pretreated dendritic cells reduce the proliferative capacity but promote interleukin-10 production in ovalbumin-specific T cells. (A) Modulatory effects of Dll4-pretreated dendritic cells (DCs) on T cell proliferation. Freshly purified DO11.10 CD4^+^ T cells (2×10^5^ cells/well) were co-cultured with irradiated immature DCs, OVA-pulsed DCs, or OVA plus Dll4-treated DCs at DC/T-cell ratios of 1∶5, 1∶10, 1∶20, 1∶40 and 1∶80 in 96-well round-bottomed plates. After 3 d of culture, cells were pulsed with 1 µCi/well of [^3^H]-thymidine for 16 to 18 h. Specific incorporation of [^3^H]-thymidine was determined with a β-counter, and results are expressed as cpm. (B) Interleukin (IL)-10 and interferon (IFN)-γ production increased in T-cell lines when generated with Dll4-pretreated DCs in vitro. T-cell lines were induced using repetitive stimulation of DO11.10 CD4^+^ T cells (1×10^6^ cells/well) with immature DCs (control), OVA-activated DCs (DC/OVA), or OVA plus Dll4-treated DCs (DC/OVA-Dll4) at a DC/T-cell ratio of 2: 1 in the presence of IL-2 (10 units/mL). After 2 rounds of stimulation, T cells were collected and stimulated with irradiated DCs and the various dosages of OVA peptide (1, 2 and 4 µg/mL). Cytokine secretion was analyzed by ELISA after stimulation for 72 h. Results from 3 independent experiments are shown and presented as the mean ± SEM. *p<0.05, **p<0.01, ***p<0.001 compared to the DC/OVA group.

Mature DCs have the capacity to induce proliferation of activated T cells at much higher levels than immature DCs. To determine the ability of DCs to induce T cell proliferation, OVA-pulsed DCs were co-cultured with DO11.10 naive CD4^+^ T cells at various ratios. Interestingly, Dll4-pretreated DCs cultured in the presence of OVA significantly suppressed T cell proliferation (**Figure 5A**). To analyze the influence of Dll4-pretreatment of DCs on differentiation of naive OVA-specific T cells in vitro, 2 rounds of stimulation of T cells were performed. We found that Dll4-pretreated DCs markedly enhanced development of IL-10-producing T cells (**Figure 5B**). Additionally, this T-cell line expressed slightly higher levels of IFN-γ than that observed under OVA stimulation of untreated DCs. In contrast, Dll4-pretreated DCs induced significantly lower levels of IL-4 release by in the responding T-cell line. However, Dll4-pretreated DCs did not affect the production of IL-5 by the OVA-specific T-cell line, compared to untreated DCs.

### Dll4-pretreated DCs Reduce Serum Anti-OVA IgE and Enhance Anti-OVA IgG_2a_ Levels in an Animal Model of Asthma

**Figure 6:**
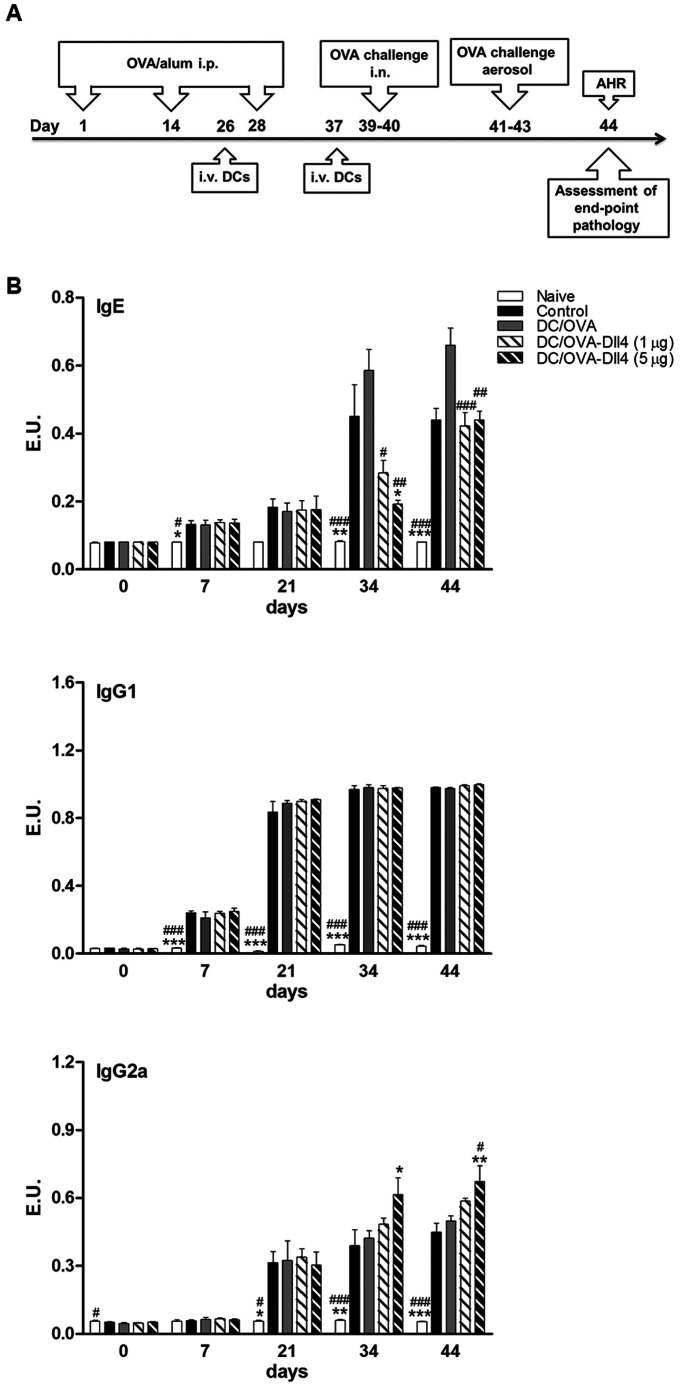
Anti-ovalbumin antibody expression levels of Dll4-modulated dendritic cell-treated mice. (A) Brief protocol of animal sensitization and challenge. On Days 1, 14, and 28, all groups of mice were sensitized by an intraperitoneal (i.p.) injection of OVA allergen. Groups of mice received either OVA-pulsed DCs (DC/OVA) or OVA plus Dll4-treated DCs [DC/OVA-Dll4 (1 µg), DC/OVA-Dll4 (5 µg)] on Days 26 and 37. Positive control mice (control) were administered PBS instead of DCs. Then mice were challenged intranasally (i.n.) with OVA on Days 39 and 40. Subsequently, mice were exposed to OVA aerosols for 3 consecutive days, and airway hyper-responsiveness (AHR) was measured 1 d after the final challenge. Bronchoalveolar lavage fluid (BALF) was collected after measuring the AHR. Negative control mice (naive) were neither sensitized with OVA nor administered DC treatment. (B) Immunoglobulin E (IgE), IgG_1_ and IgG_2a_ anti-OVA antibody expression of all groups of mice were measured using ELISA. Results are expressed as the mean ± SEM of 5 to 7 mice in each group. *p<0.05, **p<0.01 ***p<0.001 compared to the positive control group (control). ^#^ *p*<0.05, ^##^ *p*<0.01, ^###^ *p*<0.001 compared to the DC/OVA group.

To investigate the in vivo impact of Dll4-modulated DCs on the regulation of immune responses of allergic diseases, groups of mice were exposed to OVA sensitization and challenge (**Figure 6A**). These OVA-sensitized mice received injections of 1 and 5 µg/mL of Dll4-pretreated OVA-pulsed DCs (DC/OVA-Dll4 (1 µg), DC/OVA-Dll4 (5 µg)) or only OVA-pulsed DCs (DC/OVA). Control mice received PBS instead of DCs. The naive mice were neither sensitized with OVA nor administered DC treatment, although they did receive OVA challenges. Serum samples were collected on the indicated day, and OVA-specific IgE, IgG_1_ and IgG_2a_ serum antibody titers were determined by ELISA. In the control and DC/OVA groups, strong IgE and IgG_1_ production was induced (**Figure 6B**). Compared to the control and DC/OVA groups, DC/OVA-Dll4 (5 µg) treatment efficiently inhibited OVA-specific IgE production on Day 34; however, this suppressive effect was attenuated after the mice received OVA challenges. Additionally, although the administration of DC/OVA-Dll4 did not downregulate IgG_1_ production, the IgG_2a_ synthesis was slightly upregulated compared with the control and DC/OVA groups.

### Dll4-pretreated DCs Decrease the Severity of AHR and Suppress the Production of Inflammatory Mediators in BALF

**Figure 7:**
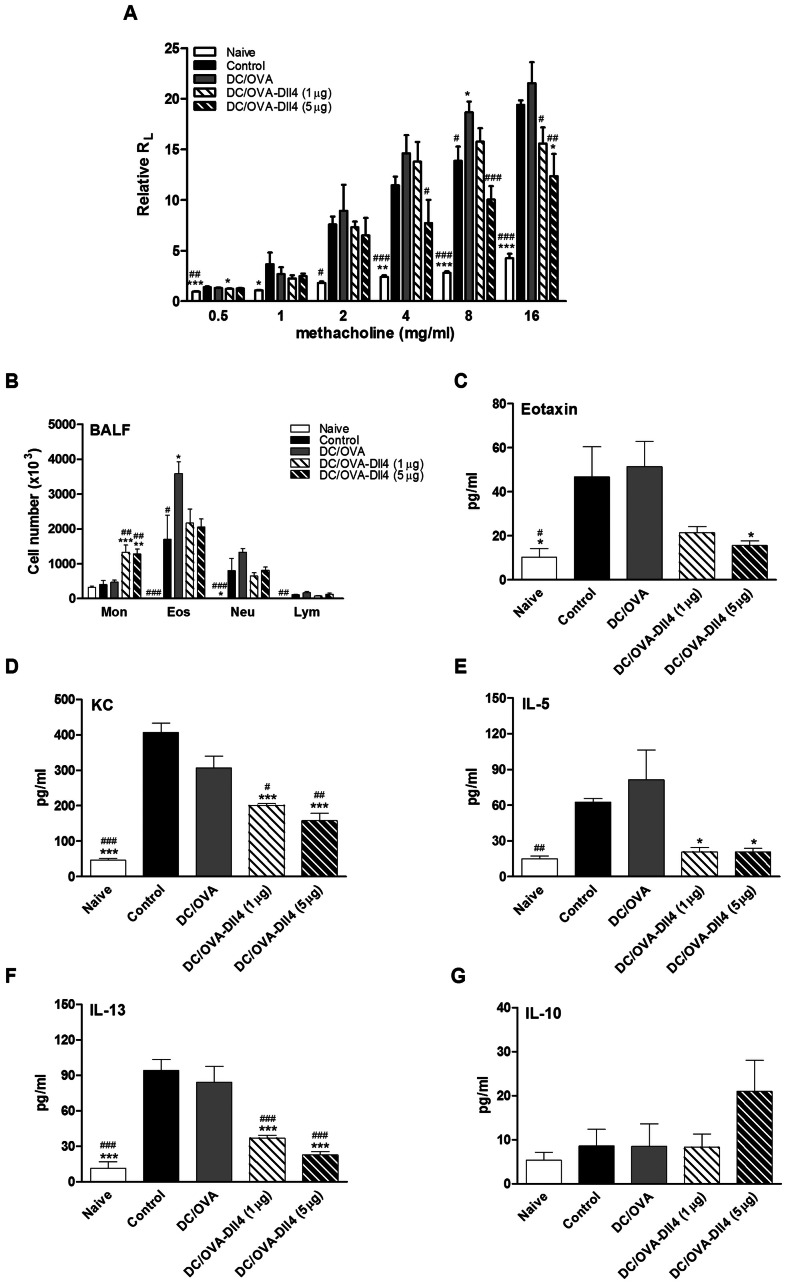
Protective effects of Dll4-modulated dendritic cells on airway hyper-responsiveness and airway inflammation. (A) Dll4-modulated dendritic cells (DCs) suppressed the development of airway hyper-responsiveness (AHR). One day after the final OVA challenge, airway resistance was measured by invasive body plethysmography. Results are expressed as the mean ± SEM of the pulmonary resistance (R_L_) in the ratio of R_L_ after PBS nebulization. (B) Changes in the cellular composition of bronchoalveolar lavage fluid (BALF) of mice exposed to OVA challenges. One day after measuring the pulmonary function parameters, each group of mice was sacrificed, and BALF was collected. Cells were counted and classified as monocytes (Mon), eosinophils (Eos), neutrophils (Neu), and lymphocytes (Lym). Results are expressed as the mean ± SEM of 5 to 7 mice in each group. (C–G) Dll4-modulated DCs decreased the levels of inflammatory mediators in bronchoalveolar lavage fluid (BALF). Eotaxin, keratinocyte-derived chemokine (KC), interleukin (IL)-5, IL-13 and IL-10 levels in BALFs of various groups of mice were measured using ELISA. Results are expressed as the mean ± SEM of 5 to 7 mice per group. *p<0.05, **p<0.01, ***p<0.001 compared to the positive control group (control). ^#^ *p*<0.05, ^##^ *p*<0.01, ^###^ *p*<0.001 compared to the DC/OVA group.

To assess the immunomodulatory effects of DC/OVA-Dll4 on allergic asthma, we investigated both AHR and the accumulation of inflammatory cells in BALF. One day after the final OVA challenge, the airway responsiveness to aerosolized methacholine of each group of mice was measured. Mice treated with PBS or OVA-pulsed DCs and then sensitized with OVA developed markedly increased airway responsiveness to methacholine stimulation compared to that of naive mice, which were not sensitized but challenged with OVA (**Figure 7A**). However, DC/OVA-Dll4 treatment dose-dependently reduced the development of AHR compared to the DC/OVA group. In the control mice, exposure to OVA challenges markedly increased the number of eosinophils and neutrophils in the BALF (**Figure 7B**). The delivery of OVA-pulsed DCs further enhanced the severity of airway inflammation. The results showed that DC/OVA-Dll4 treatment could not efficaciously lower the increase in inflammatory cells. However, such treatment induced a marked increase in the number of monocytes. Nevertheless, in the preventive animal model of OVA-induced asthma, mice pretreated with DC/OVA-Dll4 showed significantly lower increases in eosinophils and neutrophils compared to the control and DC/OVA groups (**Supplementary Figure S1**). Eotaxin and KC are potent chemokines that recruit eosinophils and neutrophils, respectively. Thus, evaluation of the levels of these chemokines in BALF showed that the administration of DC/OVA-Dll4 significantly decreased both eotaxin and KC production (**Figures 7C and 7D**). Furthermore, we assessed levels of the critical Th2 cytokines, IL-5 and IL-13, in BALF. The data showed that DC/OVA-Dll4 treatment had suppressive effects on the production of Th2 cytokines compared to the control and DC/OVA groups (**Figures 7E and 7F**). Although the difference was not significant, the administration of DC/OVA-Dll4 enhanced IL-10 production in BALF (**Figure 7G**). This effect was observed more clearly in the preventive animal model (**Supplementary Figure S1**).

### Dll4-pretreated DCs Enhance IL-10 and IFN-γ Production in the Spleen

**Figure 8:**
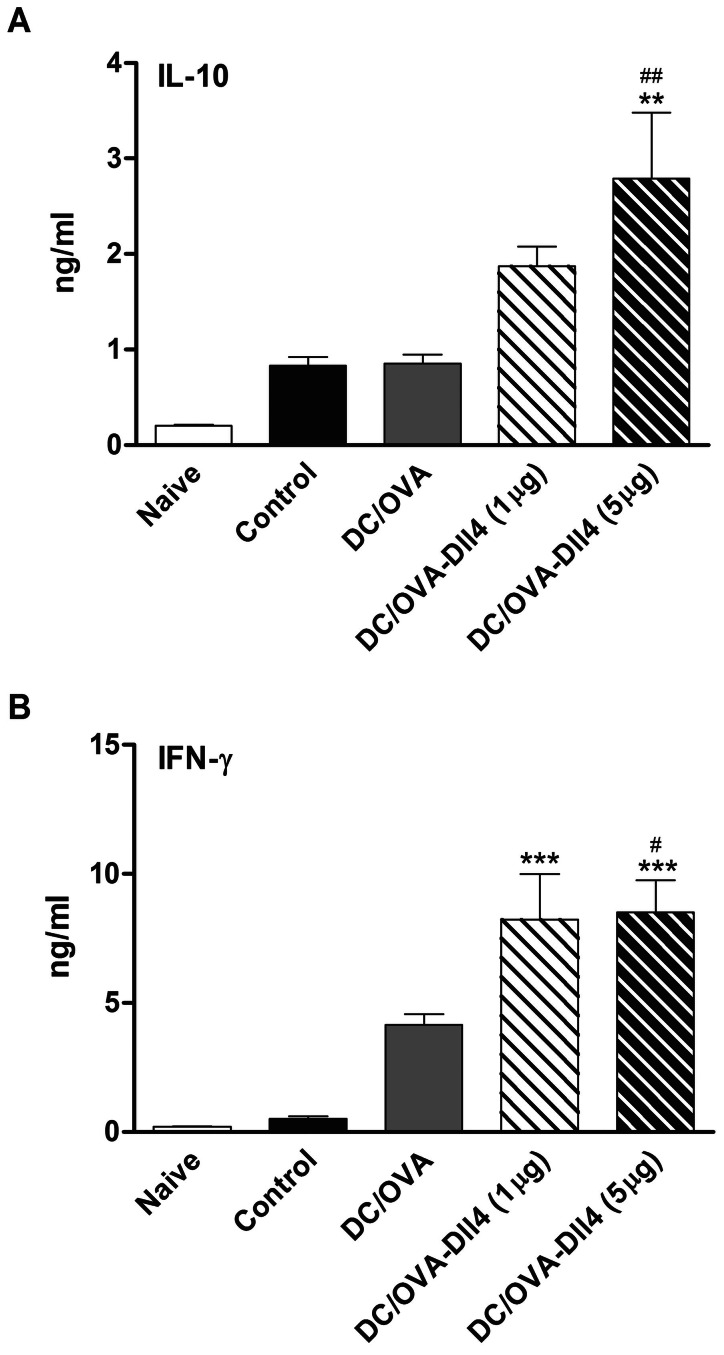
Levels of interleukin-10 and interferon-γ increase in mice receiving Dll4-modulated dendritic cells. Splenocytes (3×10^6^ cells/mL) from all groups of mice were stimulated with 100 µg/mL ovalbumin (OVA) in 24-well plates, and culture supernatants were collected after 5 d of culture. Levels of cytokine production of interleukin (IL)-10 and interferon (IFN)-γ were analyzed using ELISA. Results are expressed as the mean ± SEM of 5 to 7 mice per group. **p<0.01, ***p<0.001 compared to the positive control group (control). ^#^ *p*<0.05, ^##^ *p*<0.01 compared to the DC/OVA group.

By studying IL-10 and IFN-γ concentrations in the cell culture supernatant from the spleen, we investigated whether it was possible to influence the immune response through inducing IL-10- or IFN-γ-producing T cells. Freshly isolated spleen cells from various groups of mice were cultured with OVA. After 5 d of culture, cell supernatants were collected and analyzed using ELISA. The results showed that DC/OVA-Dll4 (5 µg) treatment markedly upregulated IL-10 production in splenocytes (**Figure 8A**). Furthermore, the data indicated that DCs treated with both doses of Dll4 exhibited a significantly enhanced level of IFN-γ production. In summary, these findings suggest that DC/OVA-Dll4 treatment might induce protective immunity against asthma through induction of IL-10 or IFN-γ production by OVA-specific T cells.

## Discussion

Notch signaling regulates a wide range of cellular development and activation events in multicellular organisms [11], [12], [16], [25]–[31]. In the past decade, research has demonstrated that Notch signaling pathways contribute to the hematopoietic and immune systems, by playing roles in the development of embryonic hematopoietic stem cells and in multiple lineage decisions of developing lymphoid and myeloid cells. Moreover, Notch signaling has been implicated as a key regulator of T cell-mediated immune responses. In recent years, clear evidence has demonstrated an essential role of Notch signaling in DC development. Cheng et al. found that incubation of hematopoietic stem cells on fibroblasts expressing Dll1 induced DC differentiation. By contrast, Jgd1-expressing fibroblasts had the opposite effect. They inhibited DC differentiation and instead promoted accumulation of immature myeloid cells [14]. Another group observed that immobilized Dll1 induced differentiation of DCs with characteristics similar to those seen in immature DCs derived from monocytes cultured with IL-4 and GM-CSF [15]. However, thus far, the role of Notch signaling in the maturation and function of DCs remains unclear. It appears that the impact of Notch signaling on DC maturation depends on the stage of DC differentiation when Notch activation is triggered, the presence of specific cytokines, and whether activation of Notch signaling is triggered by soluble or immobilized ligands. Thus, elucidating the specific conditions influencing the effect of Notch signaling on DCs could be crucial for understanding the impacts of Notch on immune responses.

In the current study, we observed that immature DCs pretreated with immobilized Dll4 exhibited increased production of IL-10 and reduced production of proinflammatory cytokines following OVA activation. A FACS analysis of DCs cultured in the presence of Dll4 plus OVA revealed upregulation of the co-stimulatory molecules, CD80 and CD86, but downregulation of MHC class II and CD40 molecules. Dll4 pretreatment upregulated Jgd1 transcripts and also enhanced Dll family mRNA synthesis in DCs following OVA activation. In addition, OVA-pulsed-Dll4-pretreated DCs exhibited an impaired ability to stimulate naive T cells in vitro. Moreover, co-culture of CD4^+^ T cells with OVA-pulsed-Dll4-modified DCs enhanced the differentiation of T cells characterized by the secretion of high levels of IL-10 and some IFN-γ. In summary, our results showed that signaling via the Dll4 ligand can induce a previously unreported maturation profile and function in DCs.

Notch ligands are composed of Dll and Jgd groups. Despite strong domain homology, it appears that signals transmitted by Dll and Jgd ligands affect target cells differently. For example, Dll4 and Jgd1 have opposing effects on angiogenesis [32], [33]. Dll4-Notch signaling downregulates expression of the vascular endothelial growth factor receptor (VEGFR) in endothelial stalk cells to inhibit vessel growth, whereas Jgd1-Notch signaling upregulates VEGFR expression in endothelial tip cells to promote vessel-distinct spatial expression patterns and opposing functional roles that regulate angiogenesis, a mechanism that might also apply to other Notch-controlled biological processes. A previous study reported that activation of Notch signaling by Jgd1 could induce activation of human DCs [21]. These DCs exhibited upregulation of maturation markers, produced IL-12, became able to activate allogenic T cells, and induced IFN-γ production. Additionally, in mouse DCs, Bugeon et al. showed that Jgd1 promoted the expression of high levels of IL-2 and TNF-α [22]. A recent report also shows that Jgd1 could antagonize Dll4 by competing for receptor binding without triggering receptor signaling [32]. Although speculative at this point, it is conceivable that the ratio between Dll4 and Jgd1 expression in microenvironments where DCs reside determines the capacity of DCs to become active or tolerogenic forms. Thus, it would be worthwhile to explore whether manipulation of different ratios of Dll4/Jgd1 in contact with DCs can alter their maturation and function.

In the current study, our results clearly indicated that intravenous injections of OVA-pulsed Dll4-treated DCs in Th2-dominant asthmatic mice significantly reduced expression levels of OVA-specific IgE, alleviated the severity of AHR, and decreased the accumulation of proinflammatory Th2 responses in the airway. This suppressive effect of DC/OVA-Dll4 treatment was more obvious in a preventive animal model of OVA-induced asthma (**Supplementary Figure S1**). How Dll4-treated DCs perform their “regulatory” role in Th2-dominatant immune response is not fully understood. A possible explanation for their negative regulatory activities is that Dll4 treatment might influence DC maturation and function. DCs are currently divided into tolerogenic immature and immunogenic mature differentiation stages, and closer inspection reveals that tolerance is observed when partial or semi-mature DCs occur. Inducers of DC semi-maturation were reported, including TNF-α, *Bordetella pertussis*, and *Lactobacilli* from the gut flora [34]–[36]. Such semi-mature DCs do not release elevated levels of proinflammatory cytokines, such as IL-1β, IL-6, TNF-α, and IL-12, and provide high levels of expression of MHC and co-stimulatory molecules [2], [37], [38]. IL-10 production by semi-mature DCs has also been described. Previous studies have demonstrated in vivo that these semi-mature DCs are actively tolerogenic by inducing IL-10^+^ CD4^+^ Treg cells in an antigen-specific manner [34]. In our studies, these OVA-activated DCs with Dll4 stimulation expressed low levels of proinflammatory cytokines and a high level of IL-10, and they were impaired in their capacity to induce T cell proliferation in vitro. Additionally, the systemic level of IL-10 in mice treated with Dll4-modulated DCs was significantly higher than that in the control mice. Therefore, we hypothesized that Dll4 converts OVA-pulsed DCs into semi-mature like antigen-presenting cells, and IL-10 may be produced by inducible Treg cells in these Dll4-modulated DC-treated mice to suppress the allergen-specific Th2 cell-mediated immune response.

Our data revealed that the drastic enhancement of IL-10 expression in the IFN-γ-producing T-cell line represents the most striking effect of Dll4-treated DCs on T cell differentiation in vitro. In an animal study, we also showed that Dll4-treated DCs had inductive effects on Th1 responses, including anti-OVA IgG_2a_ and IFN-γ production in mice. Recently, an inducible Th1-like Treg, which produces both IL-10 and IFN-γ, was reported to block the development of allergen-induced AHR in asthmatic mice [39]. A previous study also demonstrated that IL-10 produced by Th1 cells is critical in preventing immunopathologies in various infection models [40], [41] and represents a negative feedback mechanism that is independent of Treg cells. Therefore, it is tempting to speculate that Notch may play a role in these situations. We predict that inducing various levels of maturation in DCs is possible by activating the Notch signaling pathway, and the activation status of DCs is crucial for the outcome of immune responses. Dll4 may be a molecular switch between proinflammatory and anti-inflammatory functions. In an inflammatory (or danger) stage, local damaged cells may be induced to express high levels of Dll4. Thus, when DCs acquire a high density of Dll4 contact upon stimulation with an antigen, these DCs might modify the way T cells respond by selectively enhancing its IL-10 production and thereby conveying an anti-inflammatory capacity to the otherwise proinflammatory Th1 and/or Th2 response.

Numerous studies have identified the expression of Notch ligands on the surface of DCs. These ligands can cause Notch activation in T cells during interactions between DCs and T cells. In our study, we found that Dll4 treatment could induce higher expression levels of Dll4 in DCs than those of other Notch ligands. Regarding experimental airway inflammation, lymphocytes from animals treated with the anti-Dll4 antibody in vivo exhibited increased IL-5 and IL-13 production compared to the effect of the control antibody [42]. Similarly, a study demonstrated that treatment with an anti-Dll4 antibody significantly augmented the development of murine experimental allergic conjunctivitis, as measured according to Th2 cytokine production and eosinophil infiltration [43]. In addition, Schaller et al. found that Dll4 neutralization in vivo during respiratory syncytial viral infection increased Th2 cytokine production [44]. Some studies showed that Dll4 regulated the pathogenesis of allergic pulmonary disease by modulating IL-2 production [23], [45]. They demonstrated that Dll4 signaling suppresses the capacity of T cells to produce IL-2 and alter expansion of allergen-specific T cells and survival, but also reduces their development into Th2 cells. Thus, the suppressive effect of highly Dll4-expressing DCs on Th2 immunity may be part of a negative-feedback regulatory mechanism that limits exaggerated Th2 immune responses in the host and therefore modulates detrimental immune activation pathways. One study found that Treg cells expressed up to 20-fold more Dll4 than did effector T cells and may contribute to the regulation of allergic airway disease [46].

Finally, the ability to generate Dll4-modulated DCs opens new therapeutic perspectives for using semi-mature DCs in inflammatory diseases. Indeed, in vitro pulsing of Dll4-treated DCs with antigens followed by an in vivo injection could lead to differentiation of specific T cell populations able to downregulate reactivity mediated by IL-10 production. However, this therapeutic approach requires further study and characterization of specific antigens. In conclusion, we demonstrated the ability of Dll4 to induce maturation of a distinct subset of DCs that display a semi-mature phenotype after in vitro activation with OVA. In addition, these DCs induced the differentiation of IL-10- and IFN-γ-secreting T cells, and adoptive transfer of OVA-pulsed Dll4-pretreated DCs alleviated OVA-induced asthma in recipient mice. Although the available information indicates that Notch is involved in DC activation and DC-mediated T cell activation, the nature of this regulation remains largely unclear. Elucidation of the exact nature of Notch’s effects on DC maturation and functions is an arduous task but is necessary for understanding the functions of the immune system under physiological and pathological conditions.

## Supporting Information

Figure S1 **Preventive effects of Dll4-modulated dendritic cells on ovalbumin-induced asthmatic mice.** (A) Brief protocol of animal sensitization and challenge. (B) Immunoglobulin E (IgE) and IgG_2a_ anti-ovalbumin (OVA) antibody expression from OVA-immunized mice were measured by ELISA. (C) One day after the final OVA challenge, airway resistance was measured using invasive body plethysmography. (D) Cell compositions in bronchoalveolar lavage fluids (BALFs) of various groups of mice are expressed as the mean ± SEM of 5 to 7 mice per group. Cells were counted and classified as monocytes (Mon), eosinophils (Eos), neutrophils (Neu), and lymphocytes (Lym). (E) Eotaxin, keratinocyte-derived chemokine (KC), interleukin (IL)-5, IL-10 and IL-13 levels in BALFs of various groups of mice were measured using ELISA. (F) Interferon (IFN)-γ and IL-10 productions in culture supernatants of OVA-restimulated T-cells from the spleen were analyzed using ELISA. Results are expressed as the mean ± SEM of 5 to 7 mice in each group. * *p*<0.05, ** *p*<0.01, *** *p*<0.001 compared to the control group. ^#^ *p*<0.05, ^##^ *p*<0.01, ^###^ *p*<0.001 compared to the DC/OVA group.
(TIF)
