# Correction: Notch Ligand Delta-Like 4-Pretreated Dendritic Cells Alleviate Allergic Airway Responses by Enhancing IL-10 Production

**DOI:** 10.1371/journal.pone.0186529

**Published:** 2017-10-11

**Authors:** Huei-Mei Huang, George Hsiao, Chia-Kwung Fan, Chu-Lun Lin, Sy-Jye Leu, Bor-Luen Chiang, Yueh-Lun Lee

The affiliation for the seventh author is incorrect. Yueh-Lun Lee Lee is affiliated with: Department of Microbiology and Immunology, School of Medicine, College of Medicine, Taipei Medical University, Taipei, Taiwan.
