# Supplementary figures and images for: Notch Ligand Delta-Like 4-Pretreated Dendritic Cells Alleviate Allergic Airway Responses by Enhancing IL-10 Production
Source: PLoS One. 2013 May 16;8(5):e63613. doi: 10.1371/journal.pone.0063613 (PMC3656003; doi:10.1371/journal.pone.0063613)

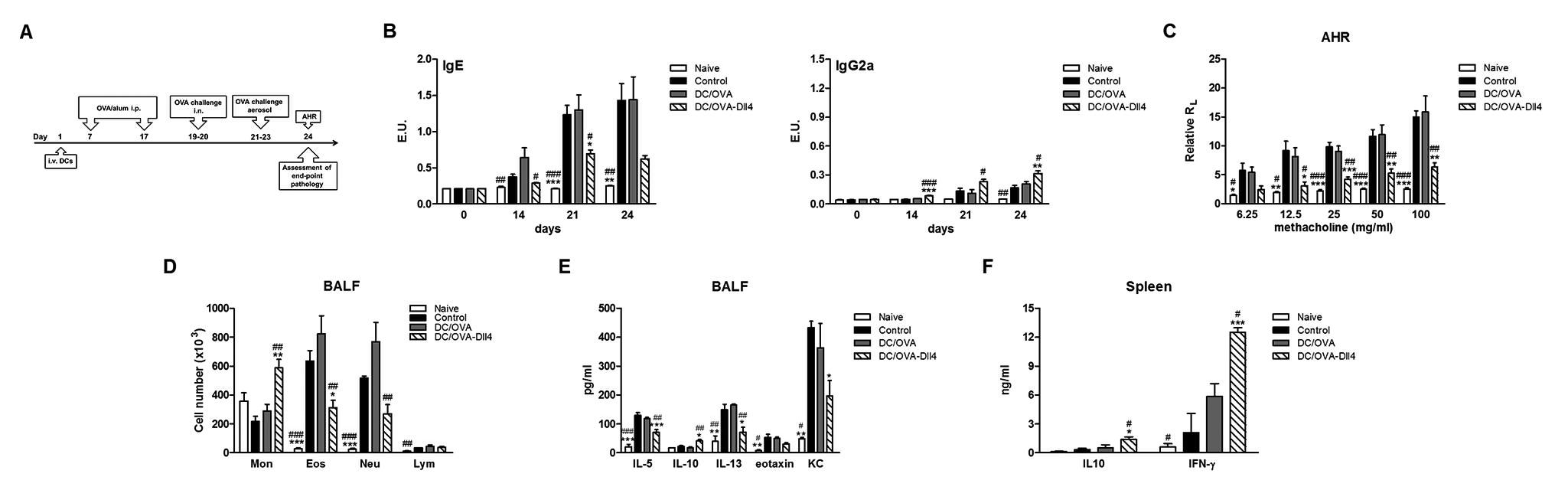

Supplement: Figure S1 — Preventive effects of Dll4-modulated dendritic cells on ovalbumin-induced asthmatic mice. (A) Brief protocol of animal sensitization and challenge. (B) Immunoglobulin E (IgE) and IgG2a anti-ovalbumin (OVA) antibody expression from OVA-immunized mice were measured by ELISA. (C) One day after the final OVA challenge, airway resistance was measured using invasive body plethysmography. (D) Cell compositions in bronchoalveolar lavage fluids (BALFs) of various groups of mice are expressed as the mean ± SEM of 5 to 7 mice per group. Cells were counted and classified as monocytes (Mon), eosinophils (Eos), neutrophils (Neu), and lymphocytes (Lym). (E) Eotaxin, keratinocyte-derived chemokine (KC), interleukin (IL)-5, IL-10 and IL-13 levels in BALFs of various groups of mice were measured using ELISA. (F) Interferon (IFN)-γ and IL-10 productions in culture supernatants of OVA-restimulated T-cells from the spleen were analyzed using ELISA. Results are expressed as the mean ± SEM of 5 to 7 mice in each group. * p<0.05, ** p<0.01, *** p<0.001 compared to the control group. # p<0.05, ## p<0.01, ### p<0.001 compared to the DC/OVA group. (TIF) [file pone.0063613.s001.tif]
